# Navigation with Polytopes: A Toolbox for Optimal Path Planning with Polytope Maps and B-spline Curves

**DOI:** 10.3390/s23073532

**Published:** 2023-03-28

**Authors:** Ngoc Thinh Nguyen, Pranav Tej Gangavarapu, Niklas Fin Kompe, Georg Schildbach, Floris Ernst

**Affiliations:** 1 Institute for Robotics and Cognitive Systems, University of Lübeck, 23562 Lübeck, Germany; 2 Institute for Electrical Engineering in Medicine, University of Lübeck, 23562 Lübeck, Germany

**Keywords:** path planner, B-spline, Bézier, polytopes, optimization, navigation with polytopes toolbox

## Abstract

To deal with the problem of optimal path planning in 2D space, this paper introduces a new toolbox named “Navigation with Polytopes” and explains the algorithms behind it. The toolbox allows one to create a polytopic map from a standard grid map, search for an optimal corridor, and plan a safe B-spline reference path used for mobile robot navigation. Specifically, the B-spline path is converted into its equivalent Bézier representation via a novel calculation method in order to reduce the conservativeness of the constrained path planning problem. The conversion can handle the differences between the curve intervals and allows for efficient computation. Furthermore, two different constraint formulations used for enforcing a B-spline path to stay within the sequence of connected polytopes are proposed, one with a guaranteed solution. The toolbox was extensively validated through simulations and experiments.

## 1. Introduction

Motion planning is an important component of the technology stack for enabling the autonomous navigation of unmanned vehicles [1]. It involves the computation of an admissible path or trajectory from the current position/configuration of the robot to a target area/point on a given map with obstacles. The difficulty of a motion planning task depends on the particular setup and problem formulation. It may involve complications such as kinodynamic constraints, uncertainties, and dynamic obstacles. Almost all approaches currently used in robotics involve a spatial discretization of the given map, called a *grid map* or *occupancy grid*. Important planning methods comprise graph search algorithms, such as Dijkstra, A*, and variants thereof, and sampling-based methods such as rapidly exploring random trees (RRT) [2,3,4]. These have been successfully applied in various works, such as for the development of a hybrid path planning algorithm and a bio-inspired control for an omni-directional mobile robot [5], or the control of a nonholonomic vehicle in tight environments [6]. They have an important drawback, however, in that the complexity of the planning problem increases rapidly with the dimensions of the map as well as the resolution of the grid map. Moreover, the grid map is an artificial construct that may complicate the path planning problem, e.g., over large empty areas or for kinodynamic constraints, and lead to unsafe or conservative results.

For this reason, this paper touts the idea of continuous motion planning and makes several contributions toward turning it into a competitive alternative. Several algorithms are proposed for efficient continuous motion planning, including the *generation of a polytope map* and a *spline-based planner*. They are described in detail in this paper and a ready-to-use implementation is provided as a Python-based toolbox, called *Navigation with Polytopes*.

In previous work, continuous motion planners using spline-based interpolations have been combined with the standard discrete frameworks [7,8,9]. In [7], the movement of a system between two exact discrete moments was studied, which relaxed some of the stringent requirements for optimal controller design in drones. In [8,9], a standard grid map, obtained with existing mapping tools such as *gmapping* [10], was transformed into a *polytope map*, in which the feasible area was decomposed into a finite number of (convex) polytopes, called *feasible polytopes*. The polytope map allows the computation of B-spline paths that completely stay inside the feasible area and, hence, the free space of the grid map. B-splines have been chosen for their *local convexity*: Each interval is bounded by the convex hull of the local control points [8,11,12]. This leads to the simple rule that the *B-spline control boundary*, i.e., the convex hull of the B-spline control point, must be fully contained inside the feasible area of the polytope map [13,14,15]. The rule has been applied widely in the literature to solve different types of motion planning problems. For example, in [16], the authors generalize the methods for motion planning with B-spline curves for constrained flatness systems. Reference [17] proposed a path planner using a B-spline curve with an obstacle avoidance property for heavy mining vehicles while [18] introduced the solution for the same problem but for Maritime autonomous surface ships. In [19], the authors further ensured the constraints on the B-spline path’s curvature for autonomous cars.

**Figure 1:**
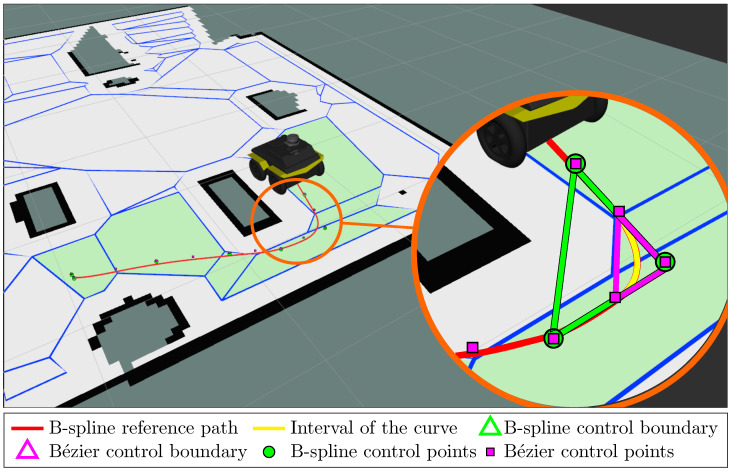
B-spline path planned within a polytope map with the *Navigation with Polytopes* (https://gitlab.rob.uni-luebeck.de/robPublic/navigation_with_polytopes, accessed on 19 February 2023) toolbox.

The approach of using B-spline parametrization, however, is conservative, as illustrated in Figure 1, which has been obtained with the *Navigation with Polytopes* toolbox (https://gitlab.rob.uni-luebeck.de/robPublic/navigation_with_polytopes, accessed on 19 February 2023). Here, the green area depicts the control boundary for the second-degree B-spline curve in red, with a portion of it highlighted in yellow. Even though the entire curve does not leave the feasible area, the control boundary is not fully contained in the feasible area. In other words, this path, despite being safe, cannot be represented with a feasible B-spline. Generally speaking, the control boundaries for B-spline control points are relatively large compared to the area covered by the curve itself.

This *conservatism* can be reduced based on prior work in the computer-aided design (CAD) community regarding the conversion between B-splines and equivalent Bézier curves [20,21]. As shown in Figure 1 with the magenta triangle, the control boundary of the corresponding Bézier curve is fully contained inside the feasible region of the polytopic map. In fact, for the same curve, the control boundary of the Bézier curve (magenta triangle) is only one-quarter the size of the original B-spline control boundary (green triangle). Thus, the usage of equivalent Bézier control points allows the design of the geometrical constraints for the computed curve to be more flexible.

A new constraint formulation for a B-spline path to stay within the feasible region of the polytope map was derived in [8]. However, the calculation of the B-spline-to-Bézier conversion parameters is not easy and is usually inefficient to compute via recursive functions, due to the original recursive formulation of the B-spline curve. For example, in [20], the authors can only derive a calculation of the conversion parameters within some middle intervals of a B-spline curve when having a sufficiently large number of control points while neglecting the rest (for more details, see Remark 1).

Sharing the line of research with the existing works [8,9] and serving as their extensions, this paper concentrates on applying two efficient tools: the polytope map of the surrounding environment and the equivalent Bézier format of a B-spline curve to solve the path planning problem for mobile robots. In particular, the following novelties are presented compared to the current state of the literature:

1. A complete procedure to construct the polytope map from a standard occupancy grid map and seek an appropriate corridor (sequence of connected polytopes), leading to the destination.
2. A new algorithm to calculate the B-spline-to-Bézier conversion matrix of a uniform B-spline curve: It takes into account the differences between each interval of the whole curve and the dependencies on the total number of control points as well as the degree of the curve.
3. New path planning constraints for a B-spline path to stay inside a sequence of connected polytopes in 2D. The equivalent Bézier representation is introduced in two variants:
  (a) Constraints that use the minimal number of control points [8];
  (b) Constraints that guarantee the existence of a valid path by providing an algebraic solution [9].
4. *Navigation with Polytopes* (https://gitlab.rob.uni-luebeck.de/robPublic/navigation_with_polytopes, accessed on 19 February 2023) toolbox: It comes as a complete Python package and serves as a framework for direct and quick implementation of existing polytope-based navigation control techniques on a realistic grid map of the environment with ROS (robot operating system) compatibility (c.f. Figure 1). It provides the following features:
  (a) Construction of a polytope map from a standard grid map with consideration of the robot’s dimension and possible noises.
  (b) Search for a sequence of connected polytopes (i.e., a polytopic corridor) connecting two given points with minimal distance.
  (c) Optimal B-spline path planning algorithm using the B-spline-to-Bézier conversion with multiple choices of algorithms [8,9].
  (d) Library for calculating and storing the B-spline-to-Bézier conversion matrix.

The remainder of the paper is organized as follows. The path planning problem and relevant details are formulated in Section 2. Next, Section 3 introduces the process of constructing a polytope map from a grid map. Section 4 introduces the notions of B-splines and its equivalent Bézier representation as well as the calculation of the B-spline-to-Bézier conversion matrix. Different path-planning constraint formulations are detailed in Section 5. Then, Section 6 introduces the *Navigation with Polytopes* toolbox. The results of the validation process using simulations and experiments are presented in Section 7 and further discussed in Section 8. Finally, Section 9 presents the conclusions and remarks on future work.

## 2. Problem Description

This paper addresses the problem of planning a 2D optimal reference path for a mobile robot to navigate between two points given the standard occupancy grid map of the surrounding environment. More specifically, the principal tool in our work is the polytope map, which describes the safety region with non-overlapping convex polytopes. It was created from the grid map via a decomposition algorithm. Within the polytope map, an appropriate sequence of connected polytopes connecting the two end-points was selected by using a graph-search algorithm. The sequence is denoted as follows:

(1) $$S≜S_{1}\cup S_{2}\cup \cdots \cup S_{q},$$

where $\left\{S_{1},\cdots ,S_{q}\right\}$ is an ordered list of q≥2 connected polytopes. Any pair of two consecutively connected polytopes $\left(S_{i},S_{i+1}\right)$ share a common edge denoted by $E_{i}$:

(2) $$E_{i}=S_{i}\cap S_{i+1}.$$

It is also assumed that the starting and ending poses $\left(P_{s},P_{f}\right)$ belong to the first and last polytopes, respectively:

(3) $$P_{s}\in S_{1},\;P_{f}\in S_{q}.$$

This allows for safe travel from $P_{s}$ to $P_{f}$ by staying inside the set S. Given the sequence polytopes, a smooth geometric path p(t) (with *t* being the curve variable, which can represent the path length, pseudo-time increment, etc.) was generated:

(4) $$p\left(t\right):\left[t_{s},t_{f}\right]\to R^{2},$$

which is required to satisfy the end-point constraints as well as the safety condition

(5) $$\begin{array}{cc} \; & p\left(t_{s}\right)=P_{s},\;p\left(t_{f}\right)=P_{f}, \end{array}$$

(6) $$\begin{array}{cc} \; & p\left(t\right)\in S,\;\forall t\in \left[t_{s},t_{f}\right]. \end{array}$$

In this work, the geometrical properties of B-spline curves are exploited (i.e., endpoint interpolation and local convexity) in order to generate a reference B-spline path satisfying the aforementioned constraints (5) and (6). Furthermore, the equivalent Bézier representation of a B-spline curve was used to reduce the conservativeness of the path planning problem. The whole planning process will be detailed sequentially throughout the rest of the paper, while the next section begins with the construction of the polytope map from a grid map.

## 3. Polytope Map

This section focuses on modeling the free space environment by describing it as a continuous polytope map. Contrary to the discrete-based occupancy grid representation, the polytope map is a continuous representation of the environment. It is defined as a list of connected 2D convex polytopes within the free space of an environment. A general convention of each polytope involves a list of ordered vertices.

### 3.1. Construction of Polytope Map from an Occupancy Grid Map

**Figure 2:**
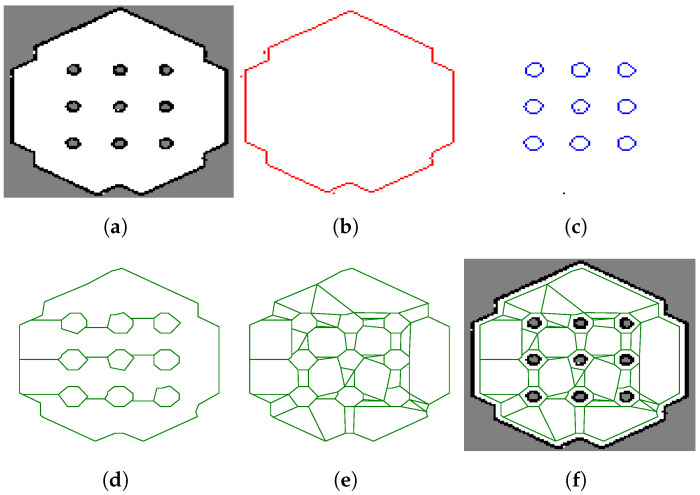
Illustration of the procedure for creating a polytope map from a standard grid map. (**a**) Occupancy grid map; (**b**) free space boundary extraction; (**c**) obstacle boundaries extraction; (**d**) free region with holes; (**e**) partition of free region into connected polytopes; (**f**) polytope map versus occupancy grid map.

This section presents an algorithm for the conversion of a standard grid map into a polytope map. The grid map can either be a binary map or a ternary representation, which is a common map used in ROS for standard navigation purposes. For example, Figure 2a shows an occupancy grid map of a simulation environment provided by ROBOTIS for the TurtleBot3 mobile robot [22]. The map is obtained by using the ROS package *gmapping* [10].

Below, one can find the Python process, which is used to construct the polytope map from a standard occupancy grid map (with a corresponding illustration on the aforementioned grid map of TurtleBot3):

1. Extract the outer boundary of the complete map using the function *findContours* with the option RETR_EXTERNAL of the OpenCV toolbox (https://opencv.org/, accessed on 19 February 2023) as shown in Figure 2b.
2. Extract the boundaries for all of the obstacles by using the same function *findContours* with the option RETR_LIST as shown in Figure 2c.
3. Simplify the contours obtained using the RDP (Ramer–Douglas–Peucker) algorithm (https://github.com/biran0079/crdp, accessed on 19 February 2023) with two parameters $\varepsilon_{rdp,o}$ for the outer boundary and $\varepsilon_{rdp,i}$ for inner obstacles [23].
4. Shrink the outer boundary and enlarge the obstacles by a safety offset $o_{p}$ by using the Gdspy toolbox (https://github.com/heitzmann/gdspy, accessed on 19 February 2023) and apply the Boolean operation to remove obstacles from the outer boundary polytope, as shown in Figure 2d.
5. Partition the obstacle-free polytope (possibly with holes) into connected polytopes by using Mark Bayazit’s algorithm (https://github.com/wsilva32/poly_decomp.py, accessed on 19 February 2023), as shown in Figure 2e.

The result of the entire procedure is the polytope map shown in Figure 2f, where it is overlaid with the original grid map. It can be seen that the free space in the environment has shrunk far from the occupied cells (i.e., obstacles) and is divided into smaller and connected polytopes. In comparison with the usage of the configuration space map in safe navigation [2], the proposed approach is slightly simpler, i.e., it simply applies an offset with the safety distance $o_{p}$ to all objects within the map. In contrast to this, the configuration space method requires calculating the Minkowski sums of the robot’s shape and the objects.

### 3.2. Finding of Appropriate Sequence of Polytopes for Navigation

**Figure 3:**
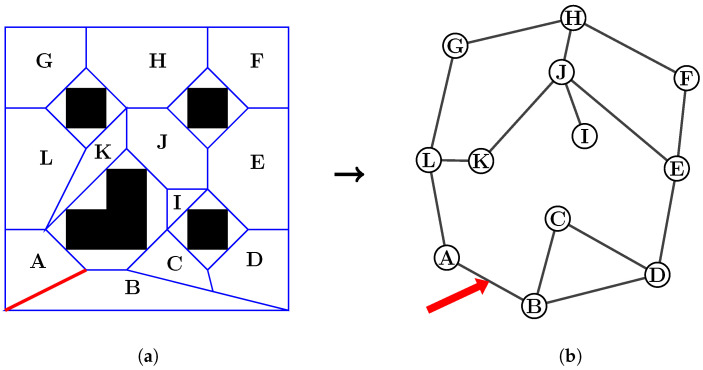
Illustration of a polytope map of an environment with four obstacles (black) and its graph representation. (**a**) Sample polytope map; (**b**) graph representation of the polytope map.

**Figure 4:**
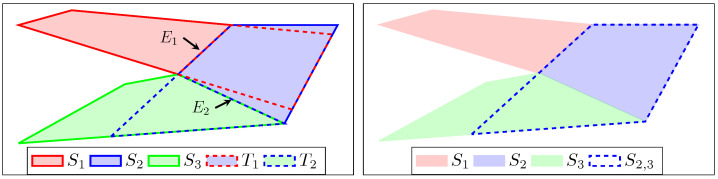
Illustration of connected polytopes, transition zones, and extended polytopes, according to Definitions 1 and 2.

After obtaining a polytope map, the next step is to find a sequence of connected polytopes (i.e., defined as an ordered list of a finite number of polytopes), which forms a corridor connecting the given initial point to the final goal. Among the sequences, two consecutive polytopes share a common edge (c.f. Figure 3 and Figure 4). In order to find that sequence, the first step is to represent the polytope map as a graph, as shown in Figure 3, in which each polytope is a node. Two nodes are considered “connected to each other” when they share a common edge (e.g., the red edge between polytopes A and B). The connection also evaluates the distance between the two polytopes by using the Euclidean distance between their center points. Then a graph search can be performed in order to obtain the shortest sequence connecting two polytopes, which contain the start and end poses. The complete process of finding such a sequence is as follows:

1. Each pair of polytopes is examined to find out if they share a common edge. If yes, then they are recognized as a connected pair.
2. From the information, an adjacency graph is created (c.f Figure 3b), which presents all polytopes as nodes and their connections to other polytopes.
3. Then a weighted graph is created from the adjacency graph by adding the distances between the center points of any pairs of connected polytopes.
4. Next, there is a search for the starting and ending polytopes by checking which polytopes contain the points $\left(P_{s},P_{f}\right)$.
5. A graph search algorithm can then be implemented on the weighted graph to obtain the sequence of polytopes $S≜S_{1}\cup S_{2}\cup \cdots \cup S_{q}$ with minimal travel distance.

### 3.3. Transition Zone and Extended Polytope

As an intermediate step toward the full navigation task between $\left(P_{s},P_{f}\right)$, consider the problem of computing a path between two connected polytopes of the sequence S (6). In order to avoid collisions with obstacles, a so-called *transition zone* is introduced, which is a subset of the second polytope and whose union with the first polytope is convex (cf. Figure 4). Thus, a robot can travel safely from the first polytope to the second one by adding a transit at the transition zone.

**Definition 1**  (Transition zone [8,9]). *The transition zone $T_{i}$ is defined for two connected polytopes $S_{i}$ and $S_{i+1}$ from* (2) *as*

(7) $$\begin{array}{c} T_{i}=S_{i+1}\cap \left(S_{i}|E_{i}\right), \end{array}$$

*in which $E_{i}$ is the common edge as defined in* (2) *and the operation $\left(S_{i}|E_{i}\right)$ gives the (possibly unbounded) polytope formed by the half-space representation of $S_{i}$ without the constraint corresponding to the edge $E_{i}$.*

**Definition 2**  (Extended polytope [8,9]). *$S_{i,i+1}$ is defined as the extension of the polytope $S_{i}$ toward the polytope $S_{i+1}$:*

(8) $$S_{i,i+1}=S_{i}\cup \;T_{i},$$

*with $T_{i}$ the transition zone defined as in* (7).

For consistency, the last extended polytope is also the last polytope, i.e., $S_{q,q+1}≜S_{q}$. Any extended polytope $S_{i,i+1}$ as defined in (8) is convex and the transition zone can also be achieved from the corresponding extended polytopes:

(9) $$T_{i}=S_{i,i+1}\cap S_{i+1}=S_{i,i+1}\cap S_{i+1,i+2}.$$

This section presents the search for the sequence of connected polytopes leading to the goal. The next section introduces an interesting path parametrization, which is called the B-spline curve, whose geometrical properties allow us to control its shape via intuitive tuning of the curve parameters and, hence, easily constrain the path to stay within a predefined sequence of connected polytopes.

## 4. B-spline and Equivalent Bézier Curves

This section presents the notions of B-spline curves and their equivalent Bézier representations. The focus is on their definitions, transformations, and further geometrical properties, while more details on both types of curves could be found in the literature [11,12,15,18,20,21]. The same notations as in some of the previous work [8,9] is used intentionally, for easy reference.

### 4.1. Definition of B-spline Curves

A *clamped uniform* B-spline curve $z\left(t\right):\left[t_{s},\;t_{f}\right]\to R^{m}$ of degree *d* is defined with *n* control points $P_{i}\in R^{m}$ (i∈{1,⋯,n}, n≥d+1) as

(10) $$z\left(t\right)=\sum_{i=1}^{n}P_{i}B_{i,d,\xi }\left(t\right)=PB_{d,\xi }\left(t\right),\;t\in \left[t_{s},t_{f}\right],$$

with $P≜\left[P_{1}\;\cdots \;P_{n}\right]\in R^{m\times n}$ gathering the control points that control the shape of the curve and needs to be defined in the path planning problem. The vector $B_{d,\xi }\left(t\right)≜{\left[B_{1,d,\xi }\left(t\right)\;\cdots \;B_{n,d,\xi }\left(t\right)\right]}^{⊤}:R\to R^{n}$ contains the B-spline basis functions of the degree *d*, whose recursive definition is given by [15,16,24]

(11) $$\begin{array}{cc} \; & B_{i,0,\xi }\left(t\right)=\left\{\begin{array}{c} 1,\;\mathrm{for}\;\tau_{i}\leq t\leq \tau_{i+1},\\ 0,\;\mathrm{otherwise}\;, \end{array}\right.\forall i\in \left\{1,\cdots ,n+d\right\}, \end{array}$$

(12) $$\begin{array}{cc} \; & B_{i,d,\xi }\left(t\right)=\frac{t-\tau_{i}}{\tau_{i+d}-\tau_{i}}B_{i,d-1,\xi }\left(t\right)+\frac{\tau_{i+d+1}-t}{\tau_{i+d+1}-\tau_{i+1}}B_{i+1,d-1,\xi }\left(t\right),\forall d\geq 1. \end{array}$$

Here, the time instances $\tau_{j}$ are *clamped* and *uniformly distributed* in a knot vector ξ:

(13) $$\begin{array}{cc} \; & \xi =\{\tau_{1}\leq \tau_{2}\leq \cdots \leq \tau_{n+d+1}\}, \end{array}$$

(14) $$\begin{array}{cc} \; & \tau_{j}=\left\{\begin{array}{cc} t_{s}, & 1\leq j\leq d,\\ t_{s}+\left(j-d-1\right)\Delta , & d+1\leq j\leq n+1,\\ t_{f}, & n+2\leq j\leq n+d+1, \end{array}\right. \end{array}$$

with $\Delta =\left(t_{f}-t_{s}\right)/\left(n-d\right)$. The *clamped* and *uniform* B-spline curve z(t) from (10) has exactly (n−d) consecutive intervals equally distributed within $\left[t_{s},t_{f}\right]$. The partial curve within the $j^{th}$ interval (j∈{1,⋯,n−d}) is given by

(15) $$z\left(j,t\right)≜z\left(t\right),\;t\in [t_{s}+\left(j-1\right)\Delta ,t_{s}+j\Delta ).$$

The B-spline curve z(t) as defined in (10)–(14) possesses the following properties:

(**P1**) The $j^{th}$ interval z(j,t) of the curve as in (15) only depends on its (d+1) neighbor control points. More specifically, z(j,t) stays within their convex hull:
  (16) $$\begin{array}{c} z\left(j,t\right)=\sum_{i=j}^{j+d}P_{i}B_{i,d,\xi }\left(t\right)\in \mathrm{Conv}\left\{P_{j}\right\}, \end{array}$$
  with $P_{j}≜\left[P_{j}\cdots P_{j+d}\right]$ containing (d+1) consecutive control points from (10).
(**P2**) The first and last control points $P_{1}$ and $P_{n}$ from (10) are also the starting and ending points of the curve z(t):
  (17) $$z\left(t_{s}\right)=P_{1},\;z\left(t_{f}\right)=P_{n}.$$
(**P3**) Derivatives of B-spline basis functions can be expressed as a linear combination of B-spline basis functions:
  (18) $$\frac{\partial B_{d,\xi }\left(t\right)}{\partial t}=M_{d,d-1}L_{d,d-1}B_{d,\xi }\left(t\right),$$
  with $B_{d,\xi }$ as in (10). The two matrices $M_{d,d-1}\in R^{n\times (n-1)}$ and $L_{d,d-1}\in R^{(n-1)\times n}$ are given in Theorems 4.1–4.3 of reference [16].

Various works in the literature have employed the aforementioned properties to adapt the B-spline framework to the problems of path/trajectory planning with obstacle avoidance and waypoint constraints. For example, in [11,14], the authors use B-splines to generate trajectories for a quadcopter system with waypoint constraints. In [13,15,18], B-spline is introduced as a general framework for obstacle and collision avoidance for more aerial vehicles. However, the local B-spline control boundary of each interval $\mathrm{Conv}\{P_{j}\}$ as in (16) is relatively large in comparison with the curve interval z(j,t) itself (c.f. Figure 1) [8,20], which causes unnecessary extra conservativeness to the motion planning problems. This problem is solved in the next section with the introduction of the equivalent Bézier representation of the B-spline curve, which provides us with a tighter local control boundary for each section of the curve.

### 4.2. Local Equivalent Bézier Representation

As proven in various works from the CAD (computer-aided design) community [20,21], any interval of a B-spline curve, as defined in (10), e.g., z(j,t) from (15), is also a Bézier curve of the same degree:

(19) $$z\left(j,t\right)=\sum_{i=1}^{d+1}{\bar{P}}_{(j-1)d+i}B_{i,d,\bar{\xi_{j}}}\left(t\right).$$

Here, ${\bar{P}}_{k}$ is the Bézier control point (k∈{(j−1)d+1,⋯,jd+1}). The formulation uses the same basis function $B_{i,d,\bar{\xi_{j}}}$ as defined in (11) and (12), but with a new knot vector $\bar{\xi_{j}}$ constructed by repeating the start and end of the interval

(20) $$\bar{\xi_{j}}=\left\{\underset{d+1\;\mathrm{knots}}{\underset{︸}{t_{s}+\left(j-1\right)\Delta ,\cdots ,t_{s}+\left(j-1\right)\Delta }},\underset{d+1\;\mathrm{knots}}{\underset{︸}{t_{s}+j\Delta ,\cdots ,t_{s}+j\Delta }}\right\},$$

with $\left(t_{s}+\left(j-1\right)\Delta ,t_{s}+j\Delta \right)$ as in (15). The Bézier control points ${\bar{P}}_{k}$ (k∈{(j−1)d+1,⋯,jd+1}) as used in (19) can be calculated from the (d+1) original B-spline control points $\left\{P_{j},\cdots ,P_{j+d}\right\}$ by using the following matrix transformation:

(21) $${\bar{P}}_{j}=P_{j}A\left(d,n,j\right).$$

Here, ${\bar{P}}_{j}≜\left[{\bar{P}}_{(j-1)d+1}\cdots {\bar{P}}_{jd+1}\right]$ and $P_{j}≜\left[P_{j}\cdots P_{j+d}\right]$ consist of (d+1) Bézier and B-spline control points, respectively. The B-spline-to-Bézier conversion matrix $A\left(d,n,j\right)\in R^{\left(d+1)\times (d+1\right)}$ is recursively defined in [20], while a new calculation method for the matrix is proposed in the next section. More interestingly, every Bézier control point is a convex combination of the B-spline control points [20]. This means that every column in the matrix A(d,n,j) adds up to 1. Since the total number of intervals is fixed at (n−d) from (15), it is possible to calculate A(d,n,j) for all j∈[1,⋯,n−d], then reformulate the transformation between the Bézier and B-spline control points as follows:

(22) $$\bar{P}=P\bar{A}\left(d,n\right),$$

with $\bar{P}≜\left[{\bar{P}}_{1}\cdots {\bar{P}}_{\bar{n}}\right]$ consisting of all the Bézier control points and P as in (10). The total number of Bézier control points needed to express the whole B-spline curve of degree *d* is

(23) $$\bar{n}=\left(n-d\right)d+1,$$

where *n* is the number of B-spline control points from (10).

As a Bézier curve is also a B-spline curve, the same properties of a local convex hull container (16) and endpoint interpolation (17) are applied to any interval of the curve. This helps to extend the *geometrical* properties (16)–(17) of the B-spline curve z(t) by applying (16) and (17) to each $j^{th}$ interval z(j,t) as in (19) of the curve for all j∈{1,⋯,n−d}:

(P1*) The $j^{th}$ interval z(j,t) stays within the convex hull of its (d+1) Bézier control points,
  (24) $$z\left(j,t\right)\in \mathrm{Conv}\left\{{\bar{P}}_{j}\right\}\subset \mathrm{Conv}\left\{P_{j}\right\},$$
  with $P_{j},{\bar{P}}_{j}$ being the B-spline and equivalent Bézier control points from (21). The convexity property (24) is significantly tighter than the standard one in (16), as proven in [21] and illustrated hereinafter.
(P2*) The B-spline curve z(t) passes through (n−d+1) waypoints, which can be determined by using only the B-spline control points (including the first and last control points as two endpoints):
  (25) $$z\left(t_{s}+\left(j-1\right)\Delta \right)={\bar{P}}_{(j-1)d+1},$$
  for all j∈{1,⋯,n−d+1}. The Bézier control points ${\bar{P}}_{(j-1)d+1}$ are actually expressed in terms of the B-spline control points (22). The proof is straightforward as $\left({\bar{P}}_{(j-1)d+1},{\bar{P}}_{(j-1)d+1}\right)$ are the two Bézier control points, which start and end the $j^{th}$ interval, respectively. Hence, they belong to the curve according to the property **P2** (17).

The next section introduces the new algorithm used for calculating the local transformation matrix A(d,n,j) from (21) for the $j^{th}$ interval and the complete matrix $\bar{A}\left(d,n\right)$ as in (22) for the whole B-spline curve.

### 4.3. Calculation of B-spline-to-Bézier Conversion Matrix

The core idea of the proposed algorithms is to consider the matrix $\bar{A}\left(d,n,j\right)$ as a variable to solve for in (21). For the predefined $j^{th}$ interval of a B-spline curve (i.e., of the degree *d* and having *n* control points), a sufficient number of sets consisting of randomly generated B-spline control points is collected together with their equivalent Bézier control points. Then, $\bar{A}\left(d,n,j\right)$ is solved by using the linear Equation (21). The process is repeated for all j∈{1,⋯,n−d}, except for some special circumstances (i.e., the repetition of values of some middle matrices as discussed in Section 4.3.3); the results are gathered into the complete transformation matrix $\bar{A}\left(d,n\right)$, as in (22). Note that the B-spline curve is formulated in an *m*-dimensional space in (10), but only 1D control points are needed to calculate the matrices. Therefore, this section is restricted to 1D points $P≜\left[P_{1}\;\cdots \;P_{n}\right]\in R^{1\times n}$ and 1D function z(t) as in (10).

#### 4.3.1. Equivalent Bézier Control Points of One Interval

It is possible to solve the equivalent Bézier control points ${\bar{P}}_{j}$ of the $j^{th}$ interval of the B-spline curve z(t) from (10) given the specific values of the B-spline control points P and the degree *d*. The idea is to uniformly sample the $j^{th}$ time interval $\left[t_{s}+\left(j-1\right)\Delta ,t_{s}+j\Delta \right)$ into (d+1) instants: ${\{}^{\left(1\right)}t_{j},\cdots {,}^{\left(d+1\right)}t_{j}\}$ (e.g., by using *linespace*) and solve the following linear equation for ${\bar{P}}_{j}$:

(26) $$B_{d,j}{\bar{P}}_{j}=\left[\begin{array}{c} z\left({}^{\left(1\right)}t_{j}\right)\\ \vdots \\ z\left({}^{\left(d+1\right)}t_{j}\right) \end{array}\right],$$

with the square matrix $B_{d,j}\in R^{\left(d+1)\times (d+1\right)}$ defined as:

$$B_{d,j}=\left[\begin{array}{cccc} \; & B_{1,d,\bar{\xi_{j}}}\left({}^{\left(1\right)}t_{j}\right) & \cdots & B_{d+1,d,\bar{\xi_{j}}}\left({}^{\left(1\right)}t_{j}\right)\\ \; & \vdots & \ddots & \vdots \\ \; & B_{1,d,\bar{\xi_{j}}}\left({}^{\left(d+1\right)}t_{j}\right) & \cdots & B_{d+1,d,\bar{\xi_{j}}}\left({}^{\left(d+1\right)}t_{j}\right) \end{array}\right].$$

#### 4.3.2. Conversion Matrix of One Interval

Next, (d+1) sets of *n* control points are randomly selected and denoted as ${}^{\left(1\right)}P,\cdots {,}^{\left(d+1\right)}P$. We further define ${}^{\left(1\right)}P_{j},\cdots {,}^{\left(d+1\right)}P_{j}$ as the control points of the $j^{th}$ interval taken from ${}^{\left(1\right)}P,\cdots {,}^{\left(d+1\right)}P$, respectively. Since the conversion matrix A(d,n,j) remains the same for different values of the control points (i.e., but not for different numbers of control points), the following equation holds true:

(27) $$\left[\begin{array}{c} {}^{\left(1\right)}P_{j}\\ \vdots \\ {}^{\left(d+1\right)}P_{j} \end{array}\right]A\left(d,n,j\right)=\left[\begin{array}{c} {}^{\left(1\right)}{\bar{P}}_{j}\\ \vdots \\ {}^{\left(d+1\right)}{\bar{P}}_{j} \end{array}\right],$$

in which ${}^{\left(i\right)}{\bar{P}}_{j}$ is calculated by using (26). Solving (27) provides the conversion matrix A(d,n,j) for the $j^{th}$ interval.

**Remark 1.**  *In [20], the conversion matrices are calculated by using a recursive definition and not by directly solving as proposed in (27). Furthermore, the calculation in [20] treats the matrix A(d,n,j) the same for all the intervals and for all control point numbers (i.e., A(d,n,j) is simplified to A(d) in [20]), which is not true. The order of j, with respect to the total number of intervals (n−d), plays an important role in the calculation; hence, the matrix needs to be considered as A(d,n,j) as in our work. For more details, the algorithm given in [20] calculates the value of A(d,n,j) only for j∈{d,⋯,n−2d+1} and n≥3d−1. It is just a subset of our general consideration of (n−d) intervals, i.e., j∈{1,⋯,n−d} and n≥d+1, which are due to the natural definition of a B-spline curve (10).*

#### 4.3.3. Conversion Matrix of the Whole Curve

Theoretically, one can repeat solving (27) for all j∈{1,⋯,n−d} with the same sets of control points ${}^{\left(1\right)}P,\cdots {,}^{\left(d+1\right)}P$ in order to obtain (n−d) conversion matrices $A\left(d,n,j\right)\in R^{\left(d+1)\times (d+1\right)}$ for (n−d) intervals. However, according to the analysis in [20], the values of the conversion matrix A(d,n,j) remain the same for j∈{d,⋯,n−2d+1} when n≥3d−1 (i.e., the domain in which the algorithm given in [20] is validated). Therefore, if our algorithm runs into these distinguished cases, it does not repeat the computation but makes use of the previously stored values.

These matrices are then stacked into the complete matrix $\bar{A}\left(d,n\right)\in R^{n\times \bar{n}}$ with $\bar{n}$ number of equivalent Bézier control points (23). The ending point of an interval is also the starting point of the next one and these points should not be repeated in the complete conversion matrix. The reader is referred to Figure 3 in reference [20] for an illustrative example of how to stack these matrices.

#### 4.3.4. Evaluation of the B-spline-to-Bézier Conversion Algorithm

**Figure 5:**
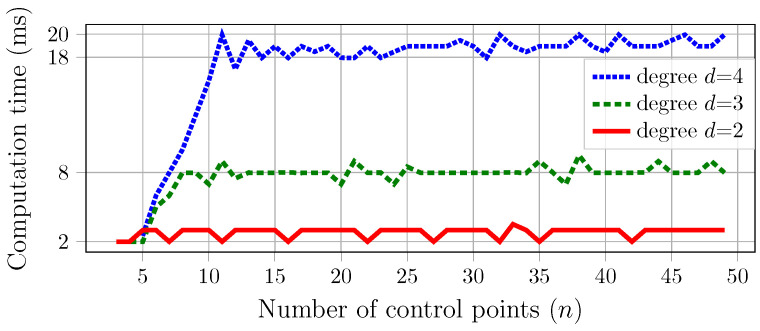
Computation time of the B-spline-to-Bézier conversion algorithm with respect to the number of control points and the curve’s degree.

Figure 5 shows the calculation time (in milliseconds) of our proposed algorithm (implemented in Python on a normal personal computer). The complete conversion matrix $\bar{A}\left(d,n\right)$ from (22) is computed with the curve degrees d∈{2,3,4} and with the number of control points *n* up to 50. It can be observed that a higher degree requires more computation time. More interestingly, when increasing the number of control points *n*, the computation time grows at the beginning but then seems to be steady. The reason is due to the special case of n≥3d−1 in which the algorithm can make use of the repeated value of the conversion matrix without a recalculation, as mentioned in Section 4.3.3. Even though the calculation time is only up to a maximum of {20,10,4} milliseconds for the {4,3,2}-degree cases, respectively, in practice, it is recommended to calculate these conversion matrices beforehand for a predicted range of *n* (e.g., up to hundreds) and a specific value of degree *d*, and then store them for online usage with real-time applications. During the online process, the calculation of $\bar{A}\left(d,n\right)$ is only performed when a new value is needed, and the result can be stored in a bank for future usage.

### 4.4. Application of B-spline-to-Bézier Conversion on 2D Path Planning

This section presents an application of the B-spline-to-Bézier conversion on 2D path planning for mobile robots in simulation. A case study of path planning in a polytopic corridor with waypoint constraints is further discussed, in which the advantages of using the B-spline-to-Bézier conversion are clearly demonstrated.

**Figure 6:**
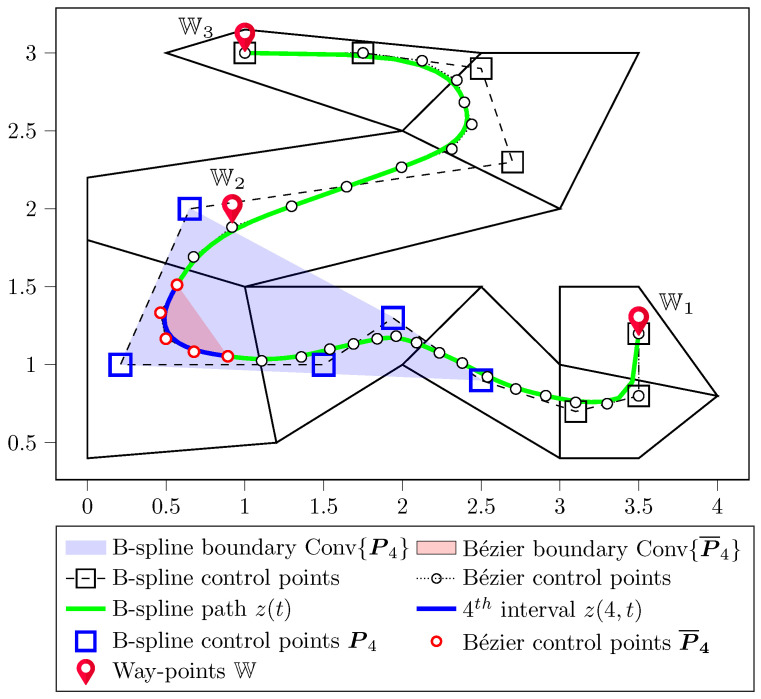
Path planning results in a polytopic corridor using fourth-order B-spline curves and a comparison between B-spline versus Bézier boundaries.

Figure 6 presents the path planning result with the B-spline-to-Bézier conversion method proposed in Section 4.3. The reference B-spline path (plotted in a solid green line) is required to stay entirely within the polytopic corridor and pass through three waypoints W={(0,3),(2,3),(0.5,2)}, which are intentionally chosen to be inside the corridor.

The first step is to convert the B-spline control points (10) to the equivalent Bézier points (22) and then apply Variant 1 introduced in Section 5.1 for placing the Bézier points, such that the curve stays inside the connected polytopes. It includes the ending point constraints, i.e., the first and last control points equal to the first and last waypoints, respectively. Finally, the extended property P2* (25) is applied to constrain the 9th Bézier control point to be the middle waypoint (2,3).

In Figure 6, the B-spline control points are plotted with square marks while the equivalent Bézier control points are plotted with circle marks. The fourth interval and its B-spline control points are highlighted in blue, which shows that the B-spline control boundary is relatively large in comparison with the curve itself and violates the safety constraint. On the other hand, the Bézier control boundary of this interval (plotted with red circle marks and filled with pink) is significantly smaller and completely stays inside the corridor. The 9th Bézier control point (marked with a red flag) is placed exactly at (2,3), which enforces the path to pass through this waypoint and, hence, satisfies all of the requirements.

The next section introduces the constraint formulations that make use of the equivalent Bézier representation for solving the path planning problem in a sequence of polytopes. The benefits of using the Bézier format over the original B-spline formulation are also highlighted via two variants of constraints.

## 5. B-spline Path Planning Algorithms in a Sequence of Connected Polytopes

This section presents our approaches for optimally placing the control points $\left\{P_{1},\cdots ,P_{n}\right\}$, such that the B-spline curve z(t), as defined in (10), satisfies all of the requirements of our path generation problem (4)–(6) and has a minimal length. For a quick summary, the curve needs to start at a point $P_{s}$, end at another point $P_{f}$, and completely stay inside the safe region S:

(28) $$z\left(t_{s}\right)=P_{s},\;z\left(t_{f}\right)=P_{f},\;z\left(t\right)\in S,$$

with $S=S_{1}\cup S_{2}\cup \cdots \cup S_{q}$ (q≥2) from (6) and $\left\{P_{s},P_{f}\right\}$ the start and end poses from (17). Hereinafter, two different formulations of constraints are introduced in order to tackle the aforementioned problems, one using a minimal number of control points and the other requiring more control points but guaranteeing the existence of a solution.

### 5.1. Variant 1: Constraint Formulation with a Minimal Number of Control Points

**Proposition 1.**  *The requirements (28) are satisfied if the following conditions are guaranteed:*

(***C1***) *Number of control points:*
(29) n=q+d.
(***C2***) *Start and end points:*
(30) $$P_{1}=P_{s},\;P_{q+d}=P_{f}.$$
(***C3***) *All the Bézier control points in one interval belong to one extended polytope (8):*
(31) $${\bar{P}}_{k}\in \;S_{j,j+1},\;\forall \;{\bar{P}}_{k}\in {\bar{P}}_{j}\;\mathrm{and}\;\forall \;j\in \left\{1,\cdots ,q\right\},$$
  *with ${\bar{P}}_{j}$ consisting of (d+1) Bézier control points given in terms of (d+1) B-spline control points $P_{j}$ as in (21); $S_{j,j+1}$ is the extended polytope as in (8).*

**Proof.**  At first, the starting and ending constraints from (28) are satisfied by condition C2 (37) due to the endpoint interpolation property (17) of the B-spline curves.

Next, by using n=d+q control points as in (29), the curve z(t) from (10) has *q* intervals. Within each interval *j*, j∈{1,⋯,q}, the following equation holds:

(32) $$z\left(j,t\right)\in \mathrm{Conv}\left\{{\bar{P}}_{j}\right\}\subseteq \;S_{j,j+1},$$

in which the convexity property is given in (24) and the latter is due to the fact that all (d+1) points in ${\bar{P}}_{j}$ stay inside $S_{j,j+1}$ as constrained by (31). The result in (32) leads to:

(33) $$z\left(t\right)\in \overset{q}{\underset{j=1}{⋃}}\;S_{j,j+1}\equiv S,\;t\in \left[t_{s},t_{f}\right].$$

This completes the proof.    □

**Remark 2.**  *Using the Bézier representation (19) allows us to formulate the constraint (31), such that it is possible to enforce “each interval z(j,t) to be inside each extended polytope” as proven in (32). This cannot be done if the original B-spline convexity property (16) is employed instead. The reason is that two consecutive B-spline boundaries share d common points (e.g., $P_{j}=\left[P_{j}\cdots P_{j+d}\right]$ and $P_{j+1}=\left[P_{j+1}\cdots P_{j+d+1}\right]$ from (16)). This leads to the fact that if the B-spline control points are employed in condition C3 (31), i.e., $P_{k}\in \;S_{j,j+1},\forall P_{k}\in P_{j},$∀j∈{1,⋯,q}, then, the following necessary condition is required:*

(34) $$\overset{j+d}{\underset{i=j}{⋂}}\;S_{i,i+1}\neq \emptyset ,\forall j\in \left\{1,\cdots ,q\right\},$$

*which is clearly not guaranteed for the extended polytopes defined in (8).*

*On the other hand, there is only one common point for the Bézier representation (24) (e.g., ${\bar{P}}_{j}$ and ${\bar{P}}_{j+1}$ share one common point ${\bar{P}}_{jd+1}$). Therefore, the necessary condition for the solution of (31) to exist is already satisfied, i.e.,*

(35) $$S_{j,j+1}\cap \;S_{j+1,j+2}\neq \emptyset ,\forall j\in \left\{1,\cdots ,q-1\right\},$$

*with $S_{j,j+1}\cap \;S_{j+1,j+2}=\;T_{j}$ as defined in (7) and (8).*

This approach exploits the property of the equivalent Bézier representation, which allows formulating the control points for each interval independently, as in (26). Therefore, it is possible to impose the constraint of “each interval within each extended polytope”, which appears to be the choice with the minimum number of control points in our analysis. However, the existence of the solution for the set of constraints (29)–(31) is not always guaranteed. This issue, unfortunately, may cause bugs and become stuck during online deployment. Therefore, we introduce another approach that requires more control points (i.e., more decision variables and heavier computation) but provides a guaranteed solution.

### 5.2. Variant 2: Constraint Formulation with Guaranteed Solution

**Proposition 2.**  *The requirements (28) are satisfied if the B-spline control points of z(t) are chosen according to the following conditions:*

(***C1***) *Number of B-spline control points:*
(36) n=d(q−1)+2,
  *which allows the curve to have d(q−2)+2 intervals as given in (15).*
(***C2***) *Start and end points:*
(37) $$P_{1}=P_{s},\;P_{n}=P_{f}.$$
  *with n as in (36).*
(***C3***) *First and last intervals stay in the first and last (i.e., $S_{q,q+1}\equiv S_{q}$) extended polytopes (8), respectively:*
(38) $${\bar{P}}_{1}\in \;S_{1,2},\;{\bar{P}}_{d(q-1)+2}\in \;S_{q,q+1},$$
(***C4**)* *and every other extended polytope contains d consecutive intervals:*
(39) $$\begin{array}{cc} {\bar{P}}_{j}\in \;S_{k,k+1}, & \forall j\in \{d(k-2)+2,\cdots ,d(k-1)+1\},\\ \; & \forall k\in \{2,\cdots ,q-1\}, \end{array}$$
  *with ${\bar{P}}_{j}$ consisting of (d+1) Bézier control points (which control the $j^{th}$ interval) given in terms of (d+1) B-spline control points $P_{j}$ as in (21) and $S_{k,k+1}$ as the extended polytope in (8).*

**Proof.**  The proof is similar to the one in Proposition 1, except for the existence of a feasible solution. Therefore, only its sketch is presented hereinafter:

(1) Condition **C2** (37) helps to ensure the start and end points of the path.
(2) Condition **C1** provides a sufficient number of intervals of the curve for the existence of a feasible solution. All of the intervals are then constrained to stay within the safe region S by two conditions **C3**–**C4** because each Bézier control boundary (i.e., the convex hull of the corresponding (d+1) Bézier control points (24)) is inside one extended polytope.
(3) Solutions for the complete problem (36)–(39) always exist. One can be found by placing the d(q−1)+2 original B-spline control points according to two conditions:
  (i) The first and last points chosen according to (37).
  (ii) Having *d* points in every transition zone $T_{k}$:
    (40) $$\begin{array}{c} \left\{P_{(k-1)d+2},\cdots ,P_{kd+1}\right\}\in T_{k},\forall k\in \left\{1,\cdots ,q-1\right\}, \end{array}$$

which is feasible since the transition $T_{k}$ is not empty for all k∈{1,⋯,q−1} as defined in (7). The next step is to prove that condition (40) ensures the satisfaction of the two conditions **C3**–**C4** (38)–(39) on the Bézier control points.

For **C3**, regarding the first interval of the curve, it is true that $P_{1}=P_{s}\in S_{1,2}$ from (37) and $\left\{P_{2},\cdots ,P_{d+1}\right\}\in T_{1}\subseteq S_{1,2}$ which ensure ${\bar{P}}_{1}\in S_{1,2}$ as $\mathrm{Conv}\left\{{\bar{P}}_{1}\right\}\subset \mathrm{Conv}\left\{P_{1}\right\}$ from (21). A similar argument is applied to the last interval of the curve; together, they lead to the satisfaction of (37).

Regarding **C4**, every extended polytope $S_{k,k+1}$ with k∈{2,⋯,q−1} contains 2d B-spline control points:

(41) $$\left\{P_{(k-2)d+2},\cdots ,P_{kd+1}\right\}\in S_{k,k+1},$$

which is due to (40) and the fact that both $T_{k}\subset S_{k,k+1}$ and $T_{k+1}\subset S_{k,k+1}$ (7)–(9). Then, for *d* consecutive intervals (k−2)d+2,⋯,(k−1)d+1, their Bézier control points satisfy:

with ${\bar{P}}_{i}$, $P_{i}$ as in (21). Note that we have $\mathrm{Conv}\left\{P_{i}\right\}\subset S_{k,k+1}$ since $P_{i}\in S_{k,k+1},\;\forall i\in \left[\left(k-2\right)d+2,\cdots ,\left(k-1\right)d+1\right]$ due to (41). Finally, the condition (39) is ensured and the resulting B-spline path satisfies all of the requirements (28). This also completes the proof.    □

**Remark 3.**  *In comparison with our approach of using a “minimal number of control points" as in (29)–(31), Proposition 2 adds d intervals to a middle polytope instead of using only one interval as in (31). It allows controlling the curve’s shape within each polytope completely and independently and, thus, always guarantee the existence of the solution. Furthermore, the feasible solution (40) is built upon the B-spline format. It is obviously only a subset of the Bézier constraints (39), while both of them can ensure the path planning requirements (28). As a result, we actually gain more feasibility and flexibility when switching to the equivalent Bézier format.*

### 5.3. Path Generation Problem with Minimal Length

This section presents the complete optimization problem used to solve the B-spline reference path satisfying the constraints (28) and minimizing the curve’s length. The property **P3** in (18) of the B-spline curve z(t) from (10) is exploited in order to formulate the length cost into a quadratic function of the control points $P_{i}\in R^{2}$, i∈{1,⋯,n} (with *n*, the number of control points, chosen as in (29) or (36)). By denoting $P≜[P_{1}\cdots P_{n}]$ from (10), the optimization problem is given by:

(42) $$\begin{array}{cc} \; & P^{*}=\arg\min_{P}\int_{t_{s}}^{t_{f}}{∥\dot{z}\left(t\right)∥}^{2}dt, \end{array}$$

subject to constraints (29)–(31) or (36)–(39) depending on the variants.

The property (18) of the B-spline curve leads to:

(43) $$\begin{array}{c} \dot{z}\left(t\right)=PM_{d,d-1}L_{d,d-1}B_{d,\xi }\left(t\right)=\sum_{i=1}^{n}Q_{i}B_{i,d,\xi }\left(t\right), \end{array}$$

with $Q_{i}\in R^{2}$ being the $i^{th}$ column of $Q=PM_{d,d-1}L_{d,d-1}\in R^{2\times 2q}$. Therefore, the optimization problem (42) is reformulated into:

(44) $$\begin{array}{cc} P^{*}=\arg\min_{P} & \sum_{i=1}^{n}\sum_{j=1}^{n}Q_{i}^{⊤}Q_{j}\int_{t_{s}}^{t_{f}}B_{i,d,\xi }\left(t\right)B_{j,d,\xi }\left(t\right)dt, \end{array}$$

subject to constraints (29)–(31) or (36)–(39) depending on the variants. Which clearly has a quadratic cost function since the integral terms are independent of the decision variables $P=[P_{1}\cdots P_{n}]$.

Finally, the reference path p(t) as required in (4)–(6) is taken as:

(45) $$p\left(t\right)=P^{*}B_{d,\xi }\left(t\right),$$

in which the optimal control points $P^{*}$ are obtained from solving the optimization problem (44) and the B-spline basis functions $B_{d,\xi }$ as used in (10) is defined with $\left[t_{s},t_{f}\right]=\left[0,1\right]$.

The theoretical background of our path planning algorithms using B-spline parametrization is complete. The next section will introduce the public repository containing the implementation of the whole path planning process and its usage guidelines.

## 6. Navigation with Polytopes Toolbox

The algorithms discussed throughout this paper were implemented in Python, published and maintained as the *Navigation with Polytopes* (https://gitlab.rob.uni-luebeck.de/robPublic/navigation_with_polytopes, accessed on 19 February 2023) toolbox. The toolbox can be used either as stand-alone scripts for research purposes or as a global path planner that is compatible with ROS (robot operating system) navigation tools. It provides a framework for the construction of a polytope map from a standard occupancy grid map, searching for an appropriate sequence of polytopes and planning a minimal-length path with different options on the B-spline or Bézier characterizations.

### 6.1. Introduction to the Toolbox

The repository of the toolbox (https://gitlab.rob.uni-luebeck.de/robPublic/navigation_with_polytopes, accessed on 19 February 2023) is organized in the following structure:

- navigation_with_polytopes—toolbox with source code.
- navigation_with_polytope_ros—integration of the toolbox into ROS.
- Examples–sample python scripts for the illustration of the toolbox.

It provides three main features:

- Constructs a polytope map from a grid map.
- Finds an appropriate sequence of polytopes.
- Plans a B-spline path with different algorithms.

**Figure 7:**
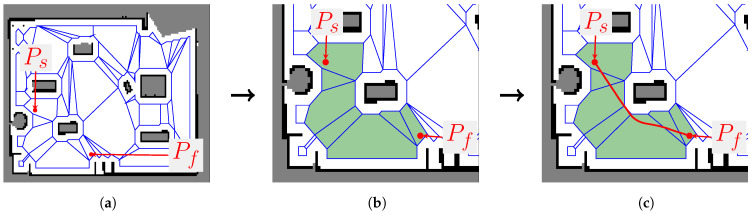
Main tasks of the *Navigation with Polytopes* toolbox. (**a**) Polytope map from a grid map; (**b**) finding a sequence of polytopes; (**c**) planning a B-spline reference path.

The outcomes of each task can be seen in Figure 7 for a given grid map. Within the scope of this paper, more details on the feature of planning the reference B-spline path using the equivalent Bézier representation are presented hereinafter. The toolbox provides the function *bspline_path_planner_polytope_map*, which receives five parameters: the starting and ending points, the polytope map, the degree *d* of the curve, and the *method*. Three options for method have been implemented as follows:

(1) *bezier_min* calls the Variant 1 algorithm given in Section 5.1, which uses the proposed B-spline-to-Bézier conversion method with a minimal number of control points [8];
(2) *bezier_guarantee* (default option) uses the Variant 2 algorithm given in Section 5.2 with a guaranteed solution [9];
(3) *bspline_guarantee* returns the algebraic solution (40) of Proposition 2. The whole calculation is done with the original B-spline format and with a guaranteed solution.

The function returns both the path defined as a list of points, and the B-spline control points P for constructing the analytical formulation z(t) (10) of the path if needed. The optimal path planning problem is implemented in Pyomo [25], Python 3, and with the solver IPOPT [26]. For ease of use, two interfaces are provided: stand-alone scripts for quick tests and easy modifications as well as a global planner package in ROS for practical usages.

### 6.2. ROS Integration

As part of the toolbox, a ROS1 package that may be used as a global path planner is also provided for convenience and integration into current projects. Two ROS nodes can be found in the ROS package *navigation with polytopes*:

- *poly_map_construct*—creates a polytope map from a given grid map by using the procedure outlined in Section 3.1.
- *bspline_path_planner_node*—given the current pose and goal, the node performs the whole path planning process. In addition to performing the tasks as the first node, it searches for the ideal sequence of polytopes and publishes the B-spline path as mentioned in Section 4.

**Figure 8:**
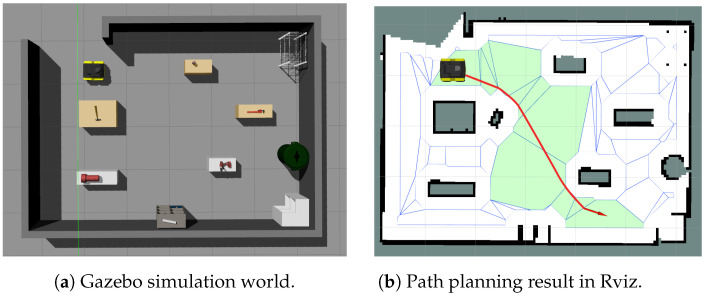
Toolbox’s result on a grid map obtained from a Gazebo simulation.

The *bspline_path_planner_node* takes several parameters for the creation of a polytope map-like robot footprint (offset $o_{p}$), RDP inner and outer epsilons, path planner parameters, such as the B-spline degree, method, etc., and parameters, such as the map frame, base frame of the robot, etc. The package provides a sample launch file, which contains all of the necessary parameters for the node. The results of the toolbox’s ROS integration are illustrated in Figure 8. The ROS package is validated using a sample environment from Gazebo, as shown in Figure 8a, and the results of the path planning algorithms from the toolbox are visualized in Rviz, as shown in Figure 8b. The polytopes (plotted in blue) are visualized in Rviz using the *jsk_ visualization* (https://github.com/jsk-ros-pkg/jsk_visualization, accessed on 19 February 2023) package. Both ROS nodes mentioned above will publish the polytope map and sequence as a msg type *jsk_ recognition_ msgs/PolygonArray* for visualization purposes in Rviz. Usage instruction and structure information (subscribed and published topics of nodes) are available in the repository.

## 7. Validation Results

The validation results of the *Navigation with Polytopes* toolbox are illustrated in this section. It firstly presents an exploration strategy for mapping an unknown environment given a top-down view figure (e.g., satellite Google Earth image). Then, the proposed path planning algorithm as well as other methods are validated in different grid maps, which are collected from realistic simulations and an actual environment. The evaluation of the toolbox when being used with ROS in various Gazebo simulation environments is presented, together with the comparisons with the default path-planning methods employed by the ROS navigation stack.

### 7.1. Exploration Strategy for Creating the Occupancy Grid Map

**Figure 9:**
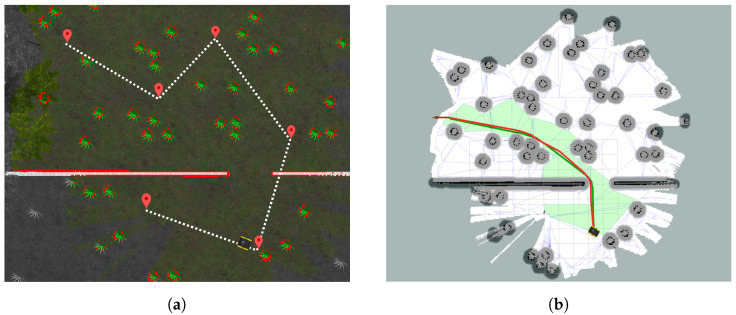
Illustration of the exploration strategy using a top-down figure of the environment and the corresponding path planning results (reference paths obtained from the *Navigation with Polytopes* toolbox and from the standard *Navfn* planner of ROS plotted in red and green lines, respectively). (**a**) Exploration program window; (**b**) path planning results after the exploration.

The proposed path planning process requires constructing a polytope map from a standard grid map. One simple method for creating such grid maps is to use the laser-based SLAM package *gmapping* while driving the robot around either manually or autonomously. We implemented an exploration program, which receives a top-down image of the environment around the robot, then allows the user to select points that will be connected as the exploration path for the robot to follow afterwards, autonomously, by using the *move_base* function in ROS (c.f. Figure 9a). Note that a simple sketch of the environment is sufficient for the program, but a screenshot of the simulation from a top-down view or satellite map image of the field is better. Moreover, the size of the map must be specified in meters and the starting position and orientation of the robot has to be specified. The exploration and mapping process is visualized in real-time in the program window as shown in Figure 9a. The dashed white line indicates the sequence in which the exploration path passes through the predefined points. Boundaries of mapped obstacles are highlighted in red, unexplored parts of the map are grayed-out and explored areas are colored. The robot’s position is marked by the robot symbol. After obtaining the grid map, one can apply the *Navigation with Polytopes* toolbox to construct the polytope map and plan a B-spline reference path (given by solid red line) as shown in Figure 9b. A comparison with the standard path planner *Navfn* in ROS (with its result plotted in a solid green line) is also presented there, which shows similarities and comparable performances (e.g., smoothness, shape, length) of the two methods. More details on the applications of the toolbox and comparisons with the *Navfn* method will be discussed in the next section.

### 7.2. Simulation Results

**Figure 10:**
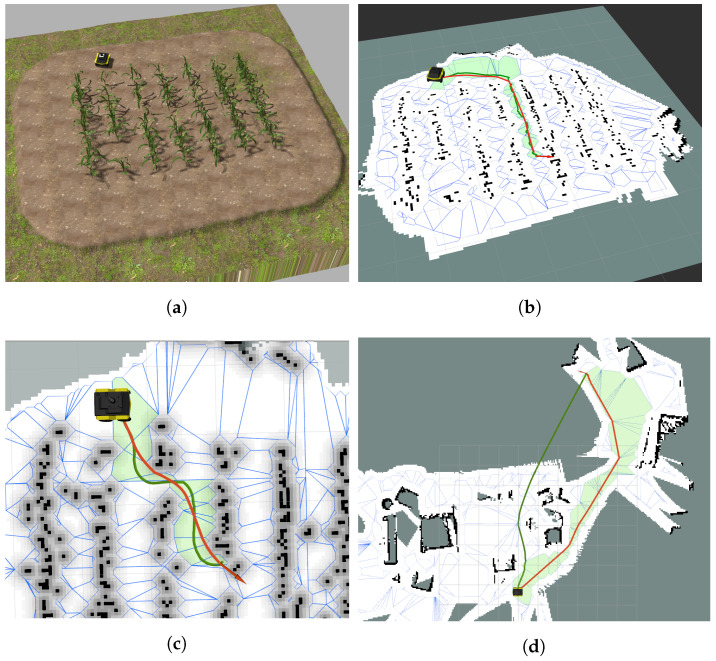
Comparisons of different path planning methods in occupancy grid maps: *Navigation with Polytopes* toolbox (red lines) versus the standard *Navfn* of ROS (green lines). (**a**) Agriculture field in the Gazebo simulation; (**b**) results in the field map: test case 1; (**c**) results in the field map: test case 2; (**d**) results in an earthquake-affected house.

**Figure A1:**
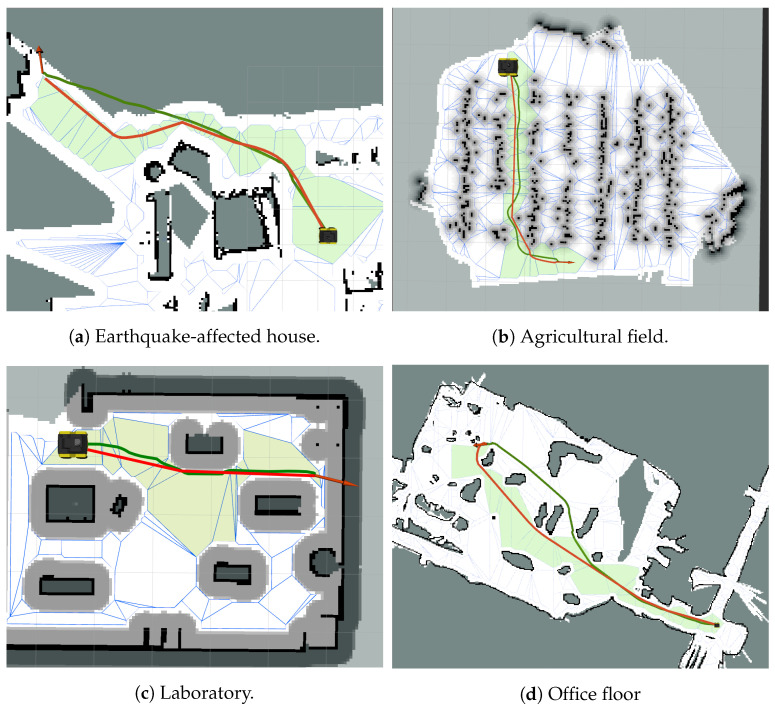
Path planning results for different occupancy grid maps; compares the performance of the *Navigation with Polytopes* toolbox (red lines) versus the standard *Navfn* in ROS (green lines).

Figure 10 shows the results of the whole path planning process performed by the toolbox on different grid maps. Figure 10a–c were captured from an agricultural field, while Figure 10d resulted from an indoor scenario after an earthquake with scattered furniture. All were simulated with high fidelity in Gazebo. Note that more results from different aspects of the aforementioned two scenarios, as well as another laboratory and office maps, are provided in Appendix A. In all scenarios, the toolbox performs the sequential steps as described throughout the paper: (i) constructing a polytope map, (ii) finding the sequence of polytopes that leads from start to goal points, and (iii) planning a reference path using one of the three methods available in the toolbox. The polytope maps are bordered in blue, the sequence of polytopes allowing safe travel from $P_{s}$ to $P_{f}$ is filled with green, and the B-spline reference path is plotted in red. The computation time for constructing the polytope map for the complex agricultural field (c.f. Figure 10a–c) is around 500 ms, while for the indoor scenario after the earthquake shown in Figure 10d, it takes up to 900 ms due to numerous small and cluttered obstacles. Next, the optimal path planning process consumes 1225, 1080, and 1460 ms for the three scenarios shown in Figure 10b, Figure 10c, and Figure 10d, respectively. Figure 9b and Figure 10b–d also present the comparison of the toolbox with the path planning results of the standard *Navfn* function of ROS. The average computation time of the *Navfn* planner is around 100 ms, which is much less than the proposed toolbox. It is understandable as the *Navfn* planner is basically a modified version of the A* algorithm [5,27], which is well-known for its fast searching capability. However, the *Navigation with Polytopes* toolbox serves as a global path planner, which only runs once at the beginning of the navigation task in order to find a safe and optimal path; spending several seconds is an acceptable trade-off with numerous advantages that the B-spline path provides in comparison with the A* path. For more details, in Figure 10d and Figure A1a, the *Navfn* path planner plans the paths through the unknown gray area, which are shorter than the B-spline paths but possibly unsafe. The reason is due to the high safety demand of the *Navigation with Polytopes* toolbox, it only considers the explored and free regions when constructing the polytope map while the *Navfn* path planner allows the movements inside the unexplored area. Furthermore, the paths resulting from the *Navfn* planner (plotted in solid green) are not as smooth as the B-spline path (in red) and are longer in most of the cases. These prove the effectiveness of the proposed minimal-length path planning algorithms in (44). Another important property of the planned paths is their smoothness. As shown in Figure 10b as well as Figure A1b–d in Appendix A, the B-spline paths (plotted in solid red lines) are significantly smoother than the results from the *Navfn* path planner (plotted in solid green lines) despite the usage of the *Savitzky–Golay* path smoother, which is already implemented in the *Navfn* planner. After extensive simulation and experimental trials, the *Navigation with Polytopes* toolbox was tested carefully with various maps of different environments and of various sizes to validate its scalability and robustness, as shown in Figure 10 and Figure A1.

## 8. Discussion

The *Navigation with Polytopes* toolbox and its theoretical background were introduced in this paper. The toolbox currently serves as a global path planner and is compatible with the ROS navigation package. It takes the standard occupancy grid map of the environment, the current robot’s position, and the selected goal as inputs and provides a safe and smooth reference path with an optimal length to the goal. Major distinctions (with respect to existing works in the literature) are the construction of a polytope map from the original grid map, the usage of the B-spline curve via its equivalent Bézier representation on the constrained path planning problem, and the conversion from B-spline to Bézier control points of the curve. As an unavoidable consequence of the optimization usage, the computation time is higher than any standard path planning methods using the grid and graph search strategies (e.g., Dijkstra, RRT [2,3,4]). However, the results obtained with the toolbox show advantages over the standard methods employed in the current path planner of the ROS navigation package, such as a shorter length, a smoother profile, and enhanced safety. Another advantage of the toolbox is that optimization can be reformulated with much flexibility. In the case of the agricultural field map (c.f. Figure 10a), the optimization problem (44) can be enforced to plan the path along the center lines of the rows and not pass through them by adding an additional constraint on the connectivity of the polytope map, i.e., by not considering two polytopes to be connected if they cut through the predefined rows. We emphasize that the use of a polytope map allows for the integration of various optimal control methods to solve different navigation problems, in addition to the primary goal of serving as a global path planner. By constructing the polytope map, the toolbox can become a framework for easily integrating existing optimal control techniques into realistic grid map data. The problems to be tackled are not limited to path/trajectory planning, but also navigation, motion control, localization, etc. The transformation of the obstacle-free space (i.e., non-occupied cells in a grid map) into a polytope map, as well as finding an appropriate polytope sequence, allows simplifying and representing a safe environment with only linear constraints (i.e., polytopic constraints). They have been employed in various optimal control applications, such as MPC (model predictive control) and mixed-integer-programming [15,28,29,30], e.g., in [28], the authors introduce an MPC controller for the safe navigation of a mobile robot within a polytope, which can push the system far away from the selected boundaries, such as walls. This controller will be added to the toolbox as the local navigation controller in the near future.

Another worthy extension would be to improve the technique of finding an appropriate sequence of polytopes by taking into account the narrowness of the corridor (i.e., only distance is counted for now). It is needed to evaluate a trade-off between a short but narrow corridor and a long but spacious one. More kinetic constraints will be taken into account, such as turning the radius and speed into the path planning algorithms, considering their effects on the solvability of the final optimization problem.

## 9. Conclusions

This paper presents the process to solve the path planning problem for a mobile robot given a standard grid map of the surrounding environment. It first constructs a polytope map of the free space and then seeks a sequence of connected polytopes leading to the goal with minimal distance. Next, a B-spline path is planned within the sequence and connects the two end points. Specifically, the B-spline path is converted into its equivalent Bézier representation in order to reduce the conservativeness of the path planning problem. Another contribution is the new technique to calculate the B-spline-to-Bézier conversion matrix, which covers all partitions of the curve. Two variants of constraints that enforce the B-spline path to stay within the aforementioned sequence of polytopes are presented with proofs. The whole procedure is implemented in Python and is publicly available as the *Navigation with Polytopes* (https://gitlab.rob.uni-luebeck.de/robPublic/navigation_with_polytopes, accessed on 19 February 2023) toolbox, which is ready to use and compatible with ROS as a global path planner for navigation.

## Data Availability

Not applicable.

## Abbreviations

The following abbreviations are used in this manuscript:

ROS	robot operating system
SLAM	simultaneous localization and mapping
CAD	computer-aided design
RDP	Ramer–Douglas–Peucker
MPC	model predictive control

## Appendix A. Illustration of the Path Planning Results from the Navigation with Polytopes Toolbox

This section provides additional examples of the path planning results obtained from the *Navigation with Polytopes* toolbox compared to the standard *Navfn* path planner in ROS. The paths from the toolbox are plotted in solid red lines, while those obtained from *Navfn* are plotted in solid green lines for four different environments: (i) an indoor scenario after an earthquake in Figure A1a, (ii) an agricultural field (cf. Figure 8a) in Figure A1b, (iii) a laboratory in Figure A1c, and (iv) an office floor in Figure A1d.
